# Making Thymus Visible: Understanding T-Cell Development from a New Perspective

**DOI:** 10.3389/fimmu.2018.00375

**Published:** 2018-03-02

**Authors:** Narges Aghaallaei, Baubak Bajoghli

**Affiliations:** ^1^Department of Hematology, Oncology, Immunology, Rheumatology and Pulmonology, University Hospital, University of Tübingen, Tübingen, Germany

**Keywords:** chemokines, thymus, imaging, medaka, zebrafish

## Abstract

T-cell development is coupled with a highly ordered migratory pattern. Lymphoid progenitors must follow a precise journey; starting from the hematopoietic tissue, they move toward the thymus and then migrate into and out of distinct thymic microenvironments, where they receive signals and cues required for their differentiation into naïve T-cells. Knowing where, when, and how these cells make directional “decisions” is key to understanding T-cell development. Such insights can be gained by directly observing developing T-cells within their environment under various conditions and following specific experimental manipulations. In the last decade, several model systems have been developed to address temporal and spatial aspects of T-cell development using imaging approaches. In this perspective article, we discuss the advantages and limitations of these systems and highlight a particularly powerful *in vivo* model that has been recently established. This model system enables the migratory behavior of all thymocytes to be studied simultaneously in a noninvasive and quantitative manner, making it possible to perform systems-level studies that reveal fundamental principles governing T-cell dynamics during development and in disease.

## Introduction

T-cell development encompasses two major migratory phases. First, in a process called thymus homing, lymphoid progenitors originating from hematopoietic sites migrate toward the thymus. Upon entry, progenitors, termed thymocytes, then migrate into distinct microenvironments and interact with thymic epithelial cells (TECs) and other non-lymphoid cells to differentiate into distinct T-cell subsets. The thymus also provides a specialized microenvironment for the selection of functional and self-tolerant T-cells. Several studies have revealed that thymic T-cell development is coupled with a highly ordered migratory pattern, called intrathymic cell migration (1–4). Both homing to the thymus and migration into distinct thymic microenvironments are tightly regulated by members of the chemokine superfamily (5–12). Chemokines are the primary proteins involved in environmental sensing and guided cell migration in many biological processes (13–16). In the thymus, TECs and other thymic resident cells, including dendritic cells (DCs), produce multiple chemokines (17). In turn, thymocytes at different developmental stages respond to these chemokines through expression of chemokine receptors. It has been shown that localization of thymocytes to a given thymic microenvironment is impeded if these cells lack a specific chemokine receptor (6, 10, 18–22). Although, these studies have greatly enhanced our current knowledge of molecular mechanisms underlying intrathymic thymocyte positioning, they have yielded only limited information regarding how cell-intrinsic mechanisms and extrinsic cues regulate thymocyte migratory behavior. The high degree of redundancy in the chemokine superfamily (i.e., several chemokine receptors can bind different chemokines and *vice versa*) and the complex chemotactic milieu of the murine thymus (4, 23) make studying thymocyte intrathymic cell trafficking challenging. It is, therefore, still not fully understood how thymocytes make directional “decisions” within distinct thymic environments and how thymocyte migratory behavior is linked to the T-cell differentiation program. Answers to these and many other fundamental questions will aid in our understanding of how T-cell development is regulated.

In this perspective article, we discuss advantages and limitations of model systems that have been established to study spatial and temporal aspects of T-cell development using imaging techniques. We then highlight a recently established transgenic fish model that allows the study of T-cell dynamics at organ, cellular, and subcellular levels.

## Visualizing Thymocyte Dynamics in Mouse Models

Imaging technology has undergone dramatic advances. It is now possible to visualize T-cell migratory behavior within secondary lymphoid tissues in live mouse models (24–28). In this setting, two-photon laser-scanning microscopy (2P microscopy) is the method of choice, because it provides large depth penetration—up to hundreds of micrometers (25). However, the murine thymus is inaccessible to 2P microscopy. This limitation can be circumvented by transplanting the thymus under the kidney capsule (29). Although, the transplanted thymus provides better access for imaging, an important consideration is that this approach requires complicated surgical procedures. Changes in parameters such as body temperature or oxygenation during the imaging procedure could also strongly influence motility parameters. Moreover, imaging of deep areas inside the thymus such as the thymic medullary region, where thymocytes undergo thymic selection, is still very technically demanding.

A widely used strategy to capture thymocyte dynamics is *ex vivo* imaging of thymic slices (30–32). In this method, fluorescently labeled thymocytes are overlaid atop vibratome-cut thymic slices several hours prior to imaging to allow these cells to migrate into their final locations (33). Thymocytes can then be monitored for a short period of time using 2P microscopy (32–34). The operating assumption providing the supporting rationale for this approach is that the structural integrity of thymic explants is maintained. One caveat is that the cytokine and chemokine milieu in thymic slices may not reflect the tissue environment that exists in live animals. Thus, the cellular behavior observed in tissue explants may not closely mimic the behavior of cells in a living organism.

## Long-Term *In Vivo* Imaging in Fish Permits the Study of Homing to the Thymus

The teleost fishes, zebrafish (*Danio rerio*) and medaka (*Oryzias latipes*), offer a number of attractive features for studying T-cell development (9, 35–38). Forward and reverse genetic approaches have been applied to characterize mechanisms underlying T-cell development in these model organisms (38–40). Apart from their strength as genetic model systems, the most important advantage of these fish is the optical access they provide. Both zebrafish and medaka embryos are transparent, offering an opportunity to directly monitor T-cell development. A range of transgenic fluorescence-based reporter lines have been developed to visualize lymphoid progenitors (41, 42), thymocyte subsets (41–45), naïve T-cells (42, 46), DCs (47), and TECs (43) in both model systems. Most of these transgenic lines carry a construct in which the expression of a fluorescent protein is under the control of cis-regulatory elements of a cell-specific gene. Alternatively, a fluorescent protein can be integrated into a gene locus using the CRISPR/Cas9 technique. We recently used this method to introduce green fluorescent protein (GFP) directly into the *autoimmune regulatory* (*aire*) gene locus to visualize the thymic medullary region in juvenile medaka (42). In fish models, fluorescent-based reporter lines have two advantages. First, they allow cells that express the gene of interest to be visualized by *in vivo* imaging. Second, they allow cells that express the fluorescent protein to be isolated using fluorescence-activated cell sorting for further characterization. This latter capability has the benefit of circumventing the lack of antibodies for T-cell surface markers in fish.

The small body size of fish larvae offers the opportunity to monitor cell trafficking at the whole-organism level using laser-scanning confocal (48) or light-sheet fluorescence microscopy (42). *In vivo* imaging of zebrafish and medaka larvae is generally noninvasive and can be continued for more than 10 h (41, 43, 49). Hence, *in vivo* imaging of transgenic zebrafish is a powerful model system for directly studying the mechanisms underlying thymus colonization by lymphoid progenitors. These studies have provided the first evidence that chemokines cooperatively regulate the migration of lymphoid progenitors toward the thymus (41). Long-term *in vivo* imaging studies have revealed that lymphoid progenitors migrate through the mesenchyme in a straight path toward the thymus (42, 43, 49). The average cell speed of thymic immigrants is ~4-fold higher than that of thymocytes located in the thymic cortical region; however, their speed drops considerably once they enter the thymic area (42). This migratory behavior is characteristic of chemotaxis, which triggers directed cell migration (13). Evidence that emerged from our study showed that simultaneous downregulation of two chemokines, Ccl25a and Cxcl12a, impedes homing to the embryonic thymus (41). Ccl25a is the only chemokine expressed in the epithelial compartment of the embryonic thymus, and Cxcl12a is expressed in cells located in the thymic periphery (41, 43). These non-overlapping expression patterns suggest that these two chemokines have non-redundant functions in guiding progenitors to the thymus. Cxcl12a is involved in transiently orienting cells toward the vicinity of the thymus, whereas Ccl25a provides the final attractive cue (43). Lymphoid precursors respond to Ccl25a and Cxcl12a through the chemokine receptors Ccr9a and Cxcr4a, respectively. Similarly, in mice, the receptor Ccr9 and its sole ligand Ccl25 are the most important factors for homing to the thymus (50–52). But this process fails in mice only when lymphoid progenitors simultaneously lack three chemokine receptors: Ccr9, Cxcr4, and Ccr7 (50). Interestingly, Ccr7 is not expressed in the medaka embryonic thymus (41, 47), while later it is expressed in the adult thymus (4 weeks post-fertilization) as revealed by RNA *in situ* hybridization (unpublished data). To what extent Ccr7 might play a role in homing to the adult thymus remains to be elusive. Overall, these studies have revealed an evolutionary functional conservation of chemokines and chemokine receptors in guiding lymphoid progenitors to settle in the thymus (9).

## *In Toto* Imaging of the Thymus in Fish Models Allows Systems-Level Studies

**Figure 1:**
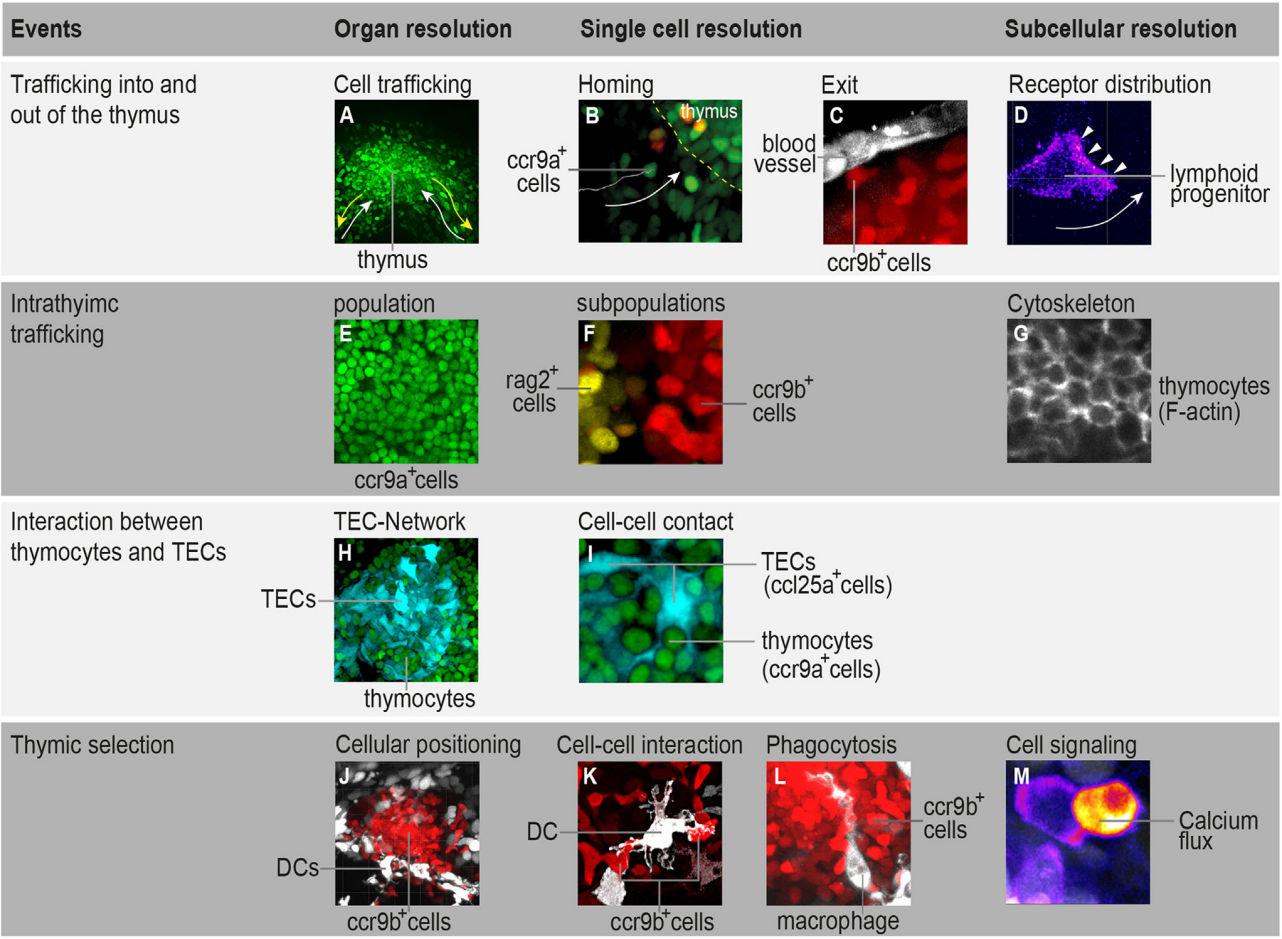
Highlighting the possibilities that *in toto* imaging of transgenic medaka fish can provide in studying spatial and temporal aspects of T-cell development. **(A)** Three-dimensional rendering of a thymus illustrating the trafficking of *ccr9a*-expressing cells (green) in the extrathymic region. White arrows indicate migration paths of thymus colonization. Yellow arrows indicate emigration paths of cells into the periphery. **(B)** Still photograph from a time-lapse recording illustrating the migration of *ccr9a*-expressing cells (green) into the thymus. Yellow dashed lines demarcate the ventral side of the thymus (*z* = 1 µm). **(C)** Still photograph from a time-lapse recording illustrating the migration of *ccr9b*-expressing mature thymocytes (red) toward a blood vessel (white). **(D)** Still photograph from a time-lapse recording illustrating the migration of a lymphoid progenitor toward the thymus. Note that thymocytes carry a green fluorescent protein (GFP) reporter fused to the Ccr9a chemokine receptor. Arrowheads indicate the accumulation of Ccr9a-GFP protein at the leading edge of the cell. Fluorescence signals are shown as a heat map. **(E)** One frame (*z* = 1 µm) from a Z-stack spanning the entire thymus **(A)** showing that the resolution of *in toto* imaging permits single thymocytes within the thymus to be distinguished. **(F)** One frame (*z* = 1 µm) from a Z-stack spanning the entire thymus illustrating the positioning of *rag2*-expressing thymocytes (yellow) in the cortex and *ccr9b*-expressing mature thymocytes (red) in the thymic medullary region. **(G)** One frame (*z* = 1 µm) from a Z-stack spanning the entire thymus of transgenic fish carrying a Lifeact reporter, a marker used to visualize F-actin (53). **(H,I)** Overview and higher magnification of a thymus in a double-transgenic [*ccl25a:tagRFP* (cyan); *ccr9a:h2b-gfp* (green)] fish. **(H)** Three-dimensional rendering of the entire thymus, illustrating the thymic epithelial cell (TEC)-network. **(I)** One frame (*z* = 1 µm) from a Z-stack spanning the entire thymus **(H)** showing that several thymocytes are in close contact with thymic epithelial cells (TECs). Note that ccl25a is expressed in TECs. **(J–L)** Overview and higher magnification of thymus in a double-transgenic [*cxcr3a:gfp* (white); *ccr9b:tagRFP* (red)] fish. **(J)** Three-dimensional rendering of the thymic medullary region showing that resident dendritic cells (DCs) are predominantly located in the interface between the thymic cortex and medullary region. **(K)** One example of the interaction of a DC (white) with a *ccr9b*-expressing mature thymocyte (red). **(L)** One example of a DC (white) engulfing a *ccr9b*-expressing thymocyte (red). **(M)** Still photograph from a time-lapse recording illustrating the rise of intracellular calcium in a thymocyte after interaction with an antigen-presenting cell. Thymocytes carry a GCaMP6s reporter for monitoring calcium level. Fluorescence signals are shown as a heat map. Information regarding transgenic reporters and imaging technique have been described previously (42).

Our current view of thymocyte population dynamics has been assembled from results obtained using several experimental systems. However, the fact that gathered data originate through various methods makes it difficult to combine them into a single model that accounts for quantitative aspects of cellular behavior. Noninvasive live imaging of the entire organ would certainly contribute to a better understanding of the relationship between cell-intrinsic mechanisms and extrinsic forces that influence thymocyte trafficking. Fish models provide such a possibility. We have recently shown that the thymus in transgenic medaka juveniles (10–12 days post-fertilization) possesses the best properties for studying thymocyte population dynamics. At this age, the thymus contains up to 10^3^ thymocytes and is composed of an outer region known as the cortex, which contains cells undergoing somatic recombination, and an inner region known as the medulla, which contains mature thymocytes undergoing negative selection (42). Time-lapse *in vivo* imaging of the entire thymus area—the so-called “*in toto* imaging”—makes it possible to determine the migratory behavior of all cells that traffic into and out of the thymus, as well as all cells that move within distinct thymic microenvironments. Therefore, *in toto* imaging of the thymus enables comparisons of cellular behaviors in different T-cell developmental contexts in a single time-lapse recording (42). The next step is the implementation of fluorescent-based reporters in this model system to elucidate core subcellular processes, such as signal transduction, transcription, protein activity, protein–protein interactions, and cytoskeletal machinery dynamics. This strategy may offer a way to transition from profiling cellular behavior to a systems-level view of the molecular principles that govern cell migration and interaction during T-cell development. Some examples of this are shown in Figure 1. Furthermore, high-content, multicolor imaging, with its high spatiotemporal resolution, has the power to support development of computational models that are close to biological reality. This opens up the possibility of predicting how changes in molecular parameters influence the migratory patterns of thymocytes. In the next two sections, we highlight how two specific topics in T-cell development—thymocyte population trafficking and thymic selection—can be addressed using *in toto* imaging of medaka transgenic models.

## Visualizing Thymocyte Population Trafficking

One important feature of the juvenile thymus is its considerably simpler chemotactic milieu (41, 42). TECs, which form a three-dimensional network, uniformly express a single chemokine, Ccl25a (41–43). On the other hand, thymocytes are spatially organized into thymic cortical and medullary regions based on the expression of two chemokine receptor paralogs, *ccr9a* and *ccr9b* (42). Time-lapse *in toto* imaging of transgenic fish carrying reporters for these two chemokine receptors has provided evidence that the migratory behavior of thymocytes is heterogeneous, lacking any discernible global pattern (42). At first glance, it seems surprising that cells with similar transcriptional levels of chemokine receptors exhibit substantially distinct migratory behaviors. However, a recently developed reporter in our laboratory revealed a different picture. In this reporter, the chemokine receptor is tagged with a fluorescent protein, a method that is routinely used to monitor receptor subcellular localization in real time. In a preliminary investigation, we observed a correlation between the abundance of chemokine receptor and the spatial position of thymocytes. Intriguingly, chemokine receptor distribution on cell membrane is highly dynamic, changing from one migration mode to another within a short period of time. The distribution of chemokine receptor on the cell surface was correlated with cell polarity and directional sensing. Bearing in mind that distinct thymic microenvironments within the medaka juvenile thymus produce the same chemokine, it is likely that cell-intrinsic mechanisms determine when a thymocyte becomes responsive to its environment. This observation strongly supports the hypothesis that the ability of cells to switch from one migration mode to another is regulated by crosstalk between chemokine receptor signaling and the cytoskeletal machinery (54).

## Visualizing Thymic Selection *In Vivo*

The formation of functional, self-tolerant T-cells in the peri-phery is mainly determined by cellular selection of thymocytes. In this process, thymocytes bearing the T-cell antigen receptor (TCR) interact with self-peptide-presenting DCs and TECs (55–57). This interaction is highly dynamic, and parameters such as the strength of TCR signaling in response to self-peptides and patterns of thymocyte motility determine the fate of thymocytes (57, 58). The ability to directly visualize this event would, therefore, provide valuable information on when, where, and how thymic selection occurs. In fish, following TCR rearrangement, the expression of the chemokine receptor *ccr9b* is induced in thymocytes, which then accumulate in the thymic medullary region (42). However, resident DCs, which play a critical role in mediating negative selection (56, 57), are predominantly localized to the interface between thymic cortical and medullary regions (42). This cellular localization remarkably resembles that of the murine thymus, in which the corticomedullary junction also contains a dense network of resident DCs (56, 57). Time-lapse *in toto* imaging in transgenic fish has revealed that thymocytes follow a novel “in-out-in” migratory pattern during negative selection. Thymocytes bearing TCRs temporarily leave the medullary region and interact with resident DCs. They remain in contact with DCs for a short period (<1 min) and then return. This migratory pattern repeats frequently within 30 min. As a consequence of this interaction, DCs can engulf and phagocytize thymocytes (42). The duration of cell–cell contact observed *in vivo* is significantly shorter than that observed *ex vivo* (58–60). To what extent these short-term and frequent contacts influence the fate of thymocytes is not yet clear. But the significance of these contacts can be evaluated by measuring TCR-induced calcium flux in thymocytes. A classical way to determine intracellular calcium levels in thymocytes is the use of specific calcium-binding fluorescent dyes (33, 58). However, genetically encoded calcium indicators (e.g., GCaMP6s) provide much better spatiotemporal resolution than chemical dyes and also enable calcium signals to be measured in specific cell types without interference from calcium signals in other cells (61). Introduction of this reporter into a transgenic model enables us to measure changes in calcium level upon cell–cell contact with high spatiotemporal resolution; an example is illustrated in Figure 1. Insight into the subcellular organization of calcium signals additionally provides essential information on cellular polarization during migration (62). The use of this reporter not only offers the benefit of determining the strength of TCR signaling in response to self-peptides, it also allows an assessment of the cellular polarity of all thymocytes prior to, during, and after negative selection.

## Selection of Model Systems and Imaging Tools

The ability to visualize thymocytes in their physiological environment represents a distinct improvement over classical methods. To date, several model systems have been established for studying dynamic aspects of T-cell development (Table 1). As we discuss here, these systems have distinct benefits, but come with certain caveats. Which model is chosen should, therefore, be based on the subject of study and optical accessibility. For example, *ex vivo* imaging of murine thymic slices is a versatile method for studying thymic selection or determining thymocyte motility in thymic cortical and medullary regions (33, 58–60, 63, 64), but it is not applicable to studying cellular trafficking into and out of the thymus. By contrast, *in vivo* imaging of transgenic zebrafish embryos enables the direct study of thymus colonization by lymphoid progenitors (41–43) as well as early events of thymopoiesis (43). On the other hand, the embryonic thymus in fish (both in zebrafish and medaka at 3–4 days post-fertilization) is not suitable for studying intrathymic cell migration, because it is not compartmentalized and is poorly populated (43). This limitation can be circumvented by imaging the thymus in juvenile medaka fish. Irrespective of the model system, selecting an appropriate microscopy system and analytical tools is equally important in assessing molecular and cellular dynamics in real time. A range of advanced light microscopy technologies are currently available with the ability to image fluorescently labeled objects at different depths and acquisition speeds (24, 27, 28). For example, 2P microscopy permits imaging cells deep inside the tissue and is currently widely used for intravital imaging in mouse models (25, 65). However, imaging of deep regions requires a longer acquisition time and, thus, decreases temporal resolution. This latter parameter is critical in precisely tracking motile cells in a densely populated tissue. Accordingly, light microscopy techniques that allow acquisition of images at very high frame rates with minimal illumination (e.g., spinning-disk confocal microscopy) are the methods of choice for simultaneously tracking the migratory behavior of all thymocytes in the medaka transgenic model system (42). High-speed imaging also has the benefit of allowing intracellular events to be monitored in real time with minimal photobleaching. These features are prerequisites for generating high-content imaging data, which provide a direct link between molecular and cellular dynamics during the recording period. One caveat is that spinning-disk confocal microscopy has a more limited tissue penetration than 2P microscopy. However, this limitation is less of an issue for *in vivo* imaging, because the thymus in fish models is located superficially, close to the skin.

**Table 1:** Utility of model systems to study different aspects of T-cell development using imaging approaches.

Subject of study	Intravital imaging of transplanted thymus	*Ex vivo* imaging of thymic explants	*In vivo* imaging of embryonic thymus^a^	*In toto* imaging of juvenile thymus^b^	Imaging techniques
Early thymopoiesis			X		LSCM
Homing to the thymus			X	X	LSCM, SDM, LSFM
Thymocyte–thymic epithelial cell interaction	X	X			2PM
			X	X	LSCM, SDM
Intrathymic cell migration	X	X			2PM
				X	SDM
Thymocytes population dynamics				X	SDM
Migratory behavior in distinct thymic microenvironments		X			2PM
				X	SDM
Positive and negative selections		X			2PM
				X	SDM, 2PM
Thymic egress			X	X	SDM

*^a^In vivo imaging of zebrafish transgenic embryos between 2.5 and 5 days post-fertilization*.

*^b^In toto imaging of the entire thymus in medaka transgenic juveniles at 10–12 days post-fertilization*.

*2PM, two-photon microscopy; LSCM, Laser scanning confocal microscopy; LSFM, light-sheet-based fluorescent microscopy; SDM, Spinning-disk microscopy*.

It is worth mentioning that apart from the fact that thymic T-cell development is remarkably evolutionary conserved in all jawed vertebrates (35, 41), there might be species-specific differences in molecular and cellular aspects that need to be considered. For example, lack of *pre-TCR*α gene would argue that pre-TCR signaling might not occur in teleost fishes (66). Therefore, future studies should be aimed at addressing the degree of functional conservation for molecules involved in cellular processes underlying T-cell development.

## Conclusion and Future Directions

With the ability to monitor molecular activity simultaneously in all thymocytes at high temporal resolution and compare cellular and subcellular dynamics in different T-cell development contexts comes the potential to generate surprising findings and formulate novel hypothesis. The simple architecture and chemokine milieu of the thymus of juvenile fish offer a novel *in vivo* platform for addressing issues that have previously been inaccessible, for example, how changes in individual molecular compounds affect organ-wide population dynamics. In addition, understanding mechanisms that underlie cellular trafficking is of clinical significance. In this latter context, fish are an ideal animal model for studying human diseases, such as T-cell acute lymphoblastic leukemia (T-ALL), which develops from the clonal expansion of malignant thymocytes. Long-term imaging of fluorescent-based reporters can be integrated into the T-ALL model to more precisely define the importance of specific parameters in the trafficking of malignant T-cells throughout the body. Moreover, these genetic tools can be used for high-throughput drug screening to precisely evaluate new drugs against specific target proteins.

## Author Contributions

NA and BB conceived and co-wrote this article.

## Conflict of Interest Statement

The authors declare that the research was conducted in the absence of any commercial or financial relationships that could be construed as a potential conflict of interest.
